# Efficacy of Periarticular Infiltration with Dexamethasone and Bupivacaine plus Adductor Canal Block Relative to That of Adductor Canal Block Alone for Patients Undergoing Total Knee Arthroplasty: A Retrospective Case-Matched Study

**DOI:** 10.1155/2023/7356192

**Published:** 2023-10-12

**Authors:** Varah Yuenyongviwat, Bunyaporn Wuttiworawanit, Nipat Panichnantho, Theerawit Hongnaparak, Khanin Iamthanaporn

**Affiliations:** Department of Orthopedics, Faculty of Medicine, Prince of Songkla University, Songkhla 90110, Thailand

## Abstract

**Purpose:**

Periarticular infiltration (PI) is a common procedure during total knee arthroplasty (TKA) for postoperative pain management. This retrospective, case-matched study aimed to evaluate the effectiveness of PI with dexamethasone and bupivacaine in combination with an adductor canal block (ACB) and compare it with that of ACB alone in reducing postoperative pain in patients with TKA.

**Methods:**

Data were collected from 66 patients who underwent TKA performed by a single surgeon. Thirty-three of them received ACB + PI, and 33 received ACB alone. However, both groups underwent identical surgical techniques and postoperative care protocols. The pain scores and fentanyl consumption of the two groups were compared.

**Results:**

The ACB + PI group had significantly lower pain scores than the ACB alone group at 8, 16, 24, and 48 hours postoperatively (*p*=0.033, 0.004, 0.038, and 0.049, respectively). The percentage of patients requiring fentanyl as a rescue medication was significantly higher for the ACB alone group (90.9%) than for the ACB + PI group (69.7%, *p*=0.03). The total fentanyl consumption was also lower for the ACB + PI group (*p* < 0.001).

**Conclusion:**

The periarticular injection of the combination of dexamethasone and bupivacaine plus ACB was more effective than ACB alone in reducing postoperative pain and fentanyl consumption in patients undergoing TKA. Further studies comparing different doses of dexamethasone or other cocktail regimens may provide additional insights into this approach.

## 1. Background

After undergoing total knee arthroplasty (TKA), managing pain effectively is crucial as it can markedly influence patient recovery and function [1]. Inadequate pain control after TKA has negative consequences, such as an increased risk of venous thromboembolism, stiff knee, development of chronic pain, and delayed recovery [2, 3]. Various pain management techniques are available for dealing with post-TKA pain, such as pharmacological usage, peripheral nerve blocks, and periarticular injection [4, 5].

The peripheral nerve block is effective in reducing pain after TKA. There are several types of peripheral nerve blocks, such as the femoral nerve block [6], sciatic nerve block [7], and adductor canal block (ACB) [8]. ACB is a type of peripheral nerve block affected by injecting a local anesthetic into the adductor canal, which is located beneath the sartorius muscle [9]. The benefit of ACB over other types of peripheral nerve blocks in TKA is the reduced risk of quadriceps weakness [10]. There is evidence in support of the efficacy of ACB for postoperative pain control after TKA [11].

Periarticular infiltration (PI) is widely performed during TKA. It involves the intraoperative injection of a local anesthetic combined with other medications, such as nonsteroidal anti-inflammatory drugs (NSAIDs) or steroids, into the soft tissue around the knee joint [12, 13]. A periarticular injection can be administered by the surgeon intraoperatively and at a low cost [14].

Several studies have compared the efficacies of PI combined with ACB and ACB alone for pain management following TKA, but the results are inconclusive [15, 16]. While some studies have found that the combined approach leads to improved postoperative pain scores and reduced morphine consumption compared to ACB alone [17, 18], others have found no significant difference between the two methods [19, 20]. In addition, the cocktail formulas used in these studies varied widely, ranging from local analgesic drugs alone to combinations with morphine or ketorolac [16–20].

However, there has been limited research on the effects of PI with dexamethasone and bupivacaine in patients who have undergone TKA and have already received ACB. Thus, the purpose of this study was to evaluate the effect of PI with dexamethasone and bupivacaine in combination with ACB on postoperative pain in patients who underwent TKA and compare it with that of ACB alone.

## 2. Methods

This was a retrospective, case-matched study that utilized data extracted from the electronic hospital database of patients who underwent primary TKA between July 2020 and June 2022. The local ethics committee and the Institutional Review Board approved this study, and the requirement for patient consent was waived.

The inclusion criteria were patients who underwent TKA for primary osteoarthritis and received a combination of spinal anesthesia and ACB. Patients with incomplete data, previous knee surgery, or pain medication protocols that did not match the study protocol were excluded. Patients who underwent TKA between July 2020 and December 2020 were categorized into the control group (patients who had ACB alone). In contrast, patients who underwent TKA between January 2021 and June 2022 and received a combination of PI and ACB were included in the experimental group.

All surgeries were performed by a single surgeon using a uniform technique (medial parapatellar approach with lateral patellar subluxation). A cemented posteriorly stabilized knee prosthesis was used. The tourniquet was inflated throughout the surgery. All patients received spinal anesthesia and ultrasound-guided ACB. However, patients in the experimental group received PI with a mixed solution of 0.5% bupivacaine 20 mL, dexamethasone 8 mg, and adrenaline 0.3 mg in saline solution in a total volume of 100 mL. Approximately 30 ml of the mixed solution was injected into the deep tissues around the medial and lateral collateral ligaments before implant insertion. After the cement was set, 40 ml of the mixture was injected into the joint capsule and muscle, 10 ml of the mixture was injected into the synovial tissue at the suprapatellar area, and 20 ml was injected at the subcutaneous and skin areas.

The same postoperative care and rehabilitation protocols were applied to all the patients. The patients received a standardized postoperative analgesic regimen consisting of oral paracetamol 500 mg every 8 hours and oral pregabalin 50 mg once daily before bedtime. Parecoxib (40 mg) was administered intravenously every 12 hours in three doses. For patients older than 65 years and those with body weights less than 55 kg, the dose was decreased to 20 mg. This was followed by a switch to oral celecoxib (400 mg) once daily. In the case of breakthrough pain, intravenous fentanyl was administered as rescue analgesia.

Following surgery, the patients were instructed to perform ankle pumping and quadriceps isometric exercises. They were provided with a supportive device to assist in walking, and range of motion exercises were introduced on the first day after the procedure. The severity of pain was assessed using a verbal numerical scale score ranging from 0 to 10, with 0 indicating no pain and 10 indicating the worst imaginable pain. A nurse evaluated the severity of pain at 4-hour intervals after surgery until the patient was discharged from the hospital.

An independent *t*-test was used to compare the patient's demographic data, including age, weight, height, body mass index (BMI), preoperative hemoglobin level, platelet count, and durations of surgery and hospital stay. The effects of sex, side of surgery, and American Society of Anesthesiologists (ASA) classification were assessed using the chi-squared test, while the pain score and fentanyl consumption were evaluated using the Wilcoxon rank-sum test. All statistical analyses were conducted using R, version 3.1.0 (R Foundation for Statistical Computing, Vienna, Austria). Statistical significance was defined as *p* ≤ 0.05.

## 3. Results

We analyzed 66 patients who met the eligibility criteria. The control group included 33 patients who only received ACB, whereas the experimental group included 33 patients who received PI and ACB.  Table 1 summarizes the demographic data of all the patients, which showed no differences between the groups.

The postoperative pain score was significantly lower in the PI group at 8, 16, 24, and 48 hours. However, there was no difference between the pain scores at 36 and 72 hours. The pain scores are presented in Table 2.

The percentage of patients who required fentanyl as a rescue medication was significantly higher for those who received ACB alone (90.9% for the ACB alone group and 69.7% for the ACB + PI group, *p*=0.03). The fentanyl consumption was lower for the patients who had ACB + PI in all timeframes (Table 3).

The duration of surgery was slightly higher for the ACB + PI group, but this was not statistically significant (84.30 ± 26.66 minutes for the ACB alone group and 91.82 ± 20.15 minutes for the ACB + PI group, *p*=0.201). However, the duration of hospital stay (from the day of surgery until discharge) was significantly lower for the ACB + PI group (4.24 ± 0.61 minutes for the ACB alone group and 3.85 ± 0.36 days for the ACB + PI group, *p*=0.02). None of the patients in either group developed a deep infection or thromboembolism or required reoperation.

## 4. Discussion

The combination of ACB and periarticular injection is widely used for pain management following TKA, and several studies have explored its effectiveness. However, research investigating the potential benefits of adding periarticular injections of dexamethasone and bupivacaine to patients who have already undergone ACB during TKA remained inconclusive [16–20]. In our study, the combination of PI and ACB was more effective in controlling postoperative pain following TKA than ACB alone.

In this study, patients who received PI had lower pain scores than those in the control group. These results are consistent with those of other studies, such as that conducted by Sawhney et al., who compared the effects of periarticular injection in patients who had received ACB with a normal saline solution containing ropivacaine, morphine, ketorolac, and normal saline alone [16]. Sawhney et al. found that patients who received periarticular injections had lower pain severity during walking, knee bending, and at rest on postoperative days 1 and 2. Similarly, Sankineani et al. reported that patients who received periarticular injections of ropivacaine, ketorolac, adrenaline, and morphine sulfate had significantly lower pain scores at 8 hours postoperatively, but no significant difference was observed at 24 and 48 hours relative to those of the control group [17]. However, some studies have reported contradictory results, such as that by Kampitak et al., who reported that patients who received periarticular injections of normal saline solution containing levobupivacaine, morphine, and adrenaline did not have different postoperative pain scores after postoperative days 1–3 [19]. These findings suggest that the effectiveness of PI may be influenced by the specific medication administered.

Our results demonstrated that patients in the periarticular injection group had lower fentanyl consumption for up to 72 hours postoperatively. Similar to a previous study that used an injection cocktail containing ropivacaine, morphine, and ketorolac, it was found that patients in the periarticular injection group used less hydromorphone on postoperative days 0 and 1 [16]. A small randomized control trial found that patients using levobupivacaine, morphine, and adrenaline had less morphine consumption for up to 6 hours after surgery than in the control group, but the overall morphine consumption of both groups at 24 and 48 hours was not significantly different [19].

The length of hospital stay is an important outcome measure in patients undergoing TKA. Our study found that patients who received periarticular injections had shorter hospital stays than those in the control group. This contradicted the reports of previous studies, including that conducted by Kampitak et al. [19] and a study using a solution of ropivacaine and adrenaline in 0.9% normal saline [18]. These studies found no significant decrease in the duration of hospital stay for the patients who received periarticular injections of a cocktail of levobupivacaine, morphine, and adrenaline relative to the control group. These conflicting results may be due to the differences in the medications used for periarticular injections. Further research is necessary to elucidate the effects of periarticular injections on the length of hospital stay after TKA.

This study has several limitations that should be acknowledged. First, this was a retrospective study; therefore, the surgeon and the evaluator were not blinded. However, all surgeries were performed by a single surgeon using an identical protocol, and the protocol for the patients was also identical, except for the addition of periarticular injection in one group. Second, we were only able to analyze pain scores at rest and were unable to assess pain during motion and range of motion because of the limited data available in our electronic database. Future prospective studies should incorporate these in their protocols. Finally, our periarticular injection cocktail contained two active substances: bupivacaine and dexamethasone. Therefore, it is difficult to determine the one with the greatest pain reduction effect after TKA. However, we hypothesized that both substances had synergistic effects on pain control.

## 5. Conclusions

Our study suggests that periarticular injections of a combination of dexamethasone and bupivacaine, in addition to ACB, are more effective than ACB alone in reducing pain and fentanyl consumption following TKA. These findings highlight the potential benefits of periarticular injections during TKA. However, further studies are needed to investigate the optimal doses of dexamethasone and bupivacaine and compare the efficacies of different cocktail regimens. Overall, our study contributes to the growing body of evidence regarding the use of periarticular injections for pain management after TKA.

## Figures and Tables

**Table 1:** Demographic data.

Characteristics	Group 1 (ACB alone) *N* = 33	Group 2 (ACB + PI) *N* = 33	P value
Gender (male: female)	2: 31	4: 29	0.392
Age (years)	66.85 ± 6.87^*∗*^	65.52 ± 6.27^*∗*^	0.413
Side (left: right)	14: 19	17: 16	0.459
Weight (kg)	66.4 ± 12.24^*∗*^	68.13 ± 11.91^*∗*^	0.562
Height (cm)	153.29 ± 7.43^*∗*^	156.35 ± 8.29^*∗*^	0.119
BMI (kg/m^2^)	28.33 ± 5.12^*∗*^	27.82 ± 4.04^*∗*^	0.66
ASA classification (I: II: III)	2: 21: 10	1: 28: 4	0.142
Preoperative hemoglobin (g/dL)	12.66 ± 1.2^*∗*^	12.85 ± 1.55^*∗*^	0.583
Platelet count (×10^3^/*µ*L)	270 ± 58.58^*∗*^	267.76 ± 61.49^*∗*^	0.88

^*∗*^Mean ± SD.

**Table 2:** Median postoperative pain score.

Time	Group 1 (ACB alone) *N* = 33	Group 2 (ACB + PI)*N* = 33	P value
8 hours	3 (0.5–4)	0 (0–3.5)	0.033
16 hours	2 (0–3)	0 (0–2)	0.004
24 hours	2 (0–3.5)	0 (0–2)	0.038
36 hours	1 (0–3)	0 (0–1)	0.084
48 hours	1 (0–3)	0 (0–2)	0.049
72 hours	0 (0–0.5)	0 (0)	0.406

**Table 3:** Median postoperative fentanyl consumption (microgram).

Time	Group 1 (ACB alone) *N* = 33	Group 2 (ACB + PI)*N* = 33	P value
0–12 hours	40 (30–65)	30 (0–30)	0.004
12–24 hours	0 (0–30)	0 (0–15)	0.108
24–48 hours	0 (0–45)	0 (0)	<0.001
48–72 hours	0 (0–30)	0 (0)	0.031
Total	70 (35–170)	30 (0–60)	<0.001

## Data Availability

The datasets generated during and/or analyzed during the current study are available from the corresponding author upon request.
